# Authors’ Reply to Petrou & Lazuras: ‘Recreational Athletes’ Use of Performance-Enhancing Substances: Results from the First European Randomized Response Technique Survey’

**DOI:** 10.1186/s40798-023-00583-7

**Published:** 2023-05-30

**Authors:** Ask Vest Christiansen, Monika Frenger, Andrea Chirico, Werner Pitsch

**Affiliations:** 1 Department of Public Health, Aarhus University, Aarhus, Denmark; 2 Institute for Sport Science, Saarland University, Saarbrücken, Germany; 3 European Institute for Socioeconomy, Saarbrücken, Germany; 4 Department of Psychology of Development and Socialization Processes, “Sapienza” University of Rome, Rome, Italy

In a letter to the editor, Michael Petrou of the Cyprus Anti-Doping Authority, and Lambros Lazuras of Sheffield Hallam University, question findings and oppose the discussion in our article “Recreational Athletes’ Use of Performance‑Enhancing Substances: Results from the First European Randomized Response Technique Survey” [1]. Petrou and Lazuras are concerned that “NADOs and other relevant stakeholders” could be misled by the article and that it could “potentially undermine the health of recreational athletes” [2].

To support their concern, Petrou and Lazuras present six objections to the article. The objections are self-contradictory and false.

1. Petrou and Lazuras highlight what they call “methodological limitations”, although they address a conceptual issue. We asked respondents if they had knowingly used prohibited substances or methods to enhance their sporting performance. Instead of applying the legal definition developed by WADA and preferred by Petrou and Lazuras, this social science pathway addresses respondents’ own understanding of “doping”.

With this approach, we obtain data on intent and known behaviour, and we avoid sky-high drop-out rates from asking respondents to familiarize themselves with the + 200 drugs on WADAs list *and* the other anti-doping rule violations set forth in Article 2.1 through Article 2.10 of the Code before responding if they had breached any of them. Petrou and Lazuras contradict themselves when they argue for using WADA’s definition while also saying that respondents may not be covered by the WADA Code and may not know what is on the list of banned substances. To add to this inconsistency, Petrou and Lazuras emphasise how psychological processes may distort respondents’ replies, yet they ignore (although they know, see next point) that the applied Randomized Response Technique with detection of Instruction Non-Compliance (INC) is structured to counter this. While we are transparent about how our prevalence data reflect respondents’ own understanding of doping, Petrou and Lazuras question this known and tested method to cast doubt on the validity of the results.

2. They seek support for their concern by reference to a 47% INC rate. However, the 47% INC was found only on the question for drug use for image enhancement, and consequently, this item was discarded for further analyses. The 47% INC has no connection to the survey’s doping question, as the INC here was 10%.
3. Petrou and Lazuras accuse us of arguing, “That doping in recreational sport is a ‘myth’”. They thereby neglect the context of our statement. We estimate an overall doping prevalence of 0.4% and a prevalence of 3.1% among males (we could not measure doping among females). Based on this, we state: “Although this is not the same as doping being absent, the idea that doping has contaminated sports at all levels is a myth.” Petrou and Lazuras thus overtly misrepresent our words.
4. A similar misrepresentation is found in the subsequent claim that we say that “National Anti-Doping Organisations (NADOs) should ‘leave recreational athletes to themselves’”. We do not! Attentive readers will realize that we discuss various possible interpretations of the estimated prevalence (p. 13). Against this background, we suggest avoiding extensive and expensive anti-doping test regimes targeting recreational athletes. In fact, we even acknowledge how educational campaigns with far-reaching potential could be valuable. It requires a conscious will to build a strawman argument for this to be equivalent to us, arguing that NADOs should “leave recreational athletes to themselves”.
5. As a leverage for the claim that our estimated doping prevalence for recreational athletes is too low, Petrou and Lazuras refer to a meta-analysis [3], which reported a 3.3% global lifetime prevalence of anabolic steroid use and 18.4% for “recreational sportspeople”. Mind that our study concerns recreational sport in general with 208 different sports represented in our data. It is not a study on the use of anabolic steroids in gyms. Criticizing our study, that concerns doping in 2019, by referring to lifetime prevalence figures for one substance group is misleading.
6. Without citing any evidence, Petrou and Lazuras conclude by saying that we “explicitly advised NADOs to disregard doping in recreational sport”. Again, this is not true.

Scientific honesty requires researchers to only draw conclusions supported by their data and results. We find an overall doping prevalence of 0.4%. It is not evident that this makes doping in recreational sport a public health issue, irrespective of what calls from policy organisations may suggest. To settle this, careful analysis of the available data and use of unbiased reasoning is required.

## Data Availability

Not applicable.

## Abbreviations

NADO: National Anti-Doping Agency
WADA: World Anti-Doping Agency
INC: Instruction Non-Compliance
